# Trends in Septicemia- and Influenza/Pneumonia-Related Mortality Among Patients With Malignant Neoplasms: A Nationwide Population-Based Study

**DOI:** 10.7759/cureus.114148

**Published:** 2026-08-07

**Authors:** Pankti K Raval, Jigarkumar S Modi, Bidhubhusan Raul, Prathusha Harikumar, Vishva Rajpuria, Deepa M, Rithik Dharan

**Affiliations:** 1 General Medicine, Nootan Medical College and Research Centre, Visnagar, IND; 2 General Medicine, Hi-Tech Medical College and Hospital, Bhubaneswar, IND; 3 General Medicine, Dhanalakshmi Srinivasan Medical College and Hospital, Perambalur, IND; 4 General Medicine, Smt. NHL (Nathiba Hargovandas Lakhmichand) Municipal Medical College, Ahmedabad, IND; 5 General Medicine, All India Institute of Medical Sciences, Madurai, Madurai, IND; 6 Pharmacology, The Tamil Nadu Dr. M.G.R. Medical University, Chennai, IND

**Keywords:** epidemiology, mortality, neoplasms, pneumonia, sepsis

## Abstract

Background: Infectious complications remain a major cause of morbidity and mortality among patients with malignant neoplasms despite advances in cancer treatment and supportive care. However, nationwide evidence on long-term trends and demographic and geographic disparities in infection-related mortality remains limited. This study aimed to evaluate the burden and temporal trends of septicemia- and influenza/pneumonia-related mortality among patients with malignant neoplasms in the United States and to examine demographic and geographic disparities and population-level predictors of infection-related mortality.

Methods: The study was conducted using a population-based ecological design with the Centers for Disease Control and Prevention's Wide-ranging Online Data for Epidemiologic Research Multiple Cause of Death Database. Deaths from 1999 to 2020 were selected based on a malignant neoplasm as the underlying cause of death and septicemia and/or influenza and pneumonia as other causes of death. The mortality burden was estimated using annual deaths and adjusted mortality rates. Time trend analysis was performed using Joinpoint regression. Demographic and geographic differences were explored with the Mann-Whitney U test, Kruskal-Wallis test, and multivariable negative binomial regression analysis.

Results: The overall mortality associated with infections had a significant reduction during the study period (Average Annual Percent Change -1.87%; 95% confidence interval -2.04 to -1.71; p < 0.001), but the rate of reduction decreased after 2012. Infections such as septicemia were responsible for a higher mortality burden and experienced a slight reduction (Average Annual Percent Change -0.38%), while influenza and pneumonia had a rapid decline in mortality (Average Annual Percent Change -3.05%). The infections led to a higher mortality rate among older adults, males, Black or African Americans, and people living in nonmetropolitan areas. Multivariable analysis identified race, male sex, and rural urbanisation as independent predictors of infection-related mortality.

Conclusions: Despite substantial reductions in infection-related mortality over two decades, septicemia remains a persistent cause of death among patients with malignant neoplasms. Persistent demographic and geographic disparities highlight the need to strengthen infection prevention, vaccination, antimicrobial stewardship, early sepsis recognition, and equitable supportive oncology care.

## Introduction

Cancer remains one of the major causes of morbidity and mortality, despite numerous breakthroughs in prevention, diagnosis, and treatment. According to estimates, about 20.6 million individuals develop cancer annually, and 9.8 million people died from this disease globally in 2024 [1]. Over the last 30 years, numerous improvements in screening, surgery, systemic therapy, immunotherapy, and supportive treatments have increased survival rates. In the United States (U.S.), the mortality rate caused by cancer has been declining for over three decades. Thus, since 1991, more than four million deaths have been avoided due to these trends. Therefore, the management of cancer patients has increasingly expanded to include not only treatment of the primary disease but also its complications, which play an important role in survival [2].

However, infectious diseases continue to be an important cause of complications in patients suffering from malignancies. Infection susceptibility is caused by the combination of the disease, immunosuppression from treatment, destruction of mucosal membranes, neutropenia, invasive procedures, and central venous catheterisation. The most important predisposing factor for infection development is neutropenia, and impairment of the innate and adaptive immune systems increases the likelihood of infection by bacteria, viruses, and fungi. Therefore, prevention and timely treatment of infections are an essential part of modern cancer treatment [3,4].

Among the complications of infections in cancer patients, sepsis and lower respiratory tract infections, especially influenza and pneumonia, contribute significantly to the mortality rate. Pneumonia is one of the most frequent severe infections in immunocompromised cancer patients, while sepsis is characterised by rapid clinical decline and high mortality [4,5]. Population studies show that pneumonia and influenza account for almost half (45.9%) of infections causing death in patients with cancer, while sepsis has a mortality risk more than twice that of the general population [6].

Although there is a growing realisation of the problem of mortality due to infections in patients suffering from malignancy, detailed population-based analyses regarding the trends, geographic variations, and predictors at the population level in this regard remain limited [4-6]. Hence, the primary objective of this study was to evaluate the burden and temporal trends of mortality from septicemia and influenza/pneumonia among patients with underlying malignant neoplasms in the U.S. between 1999 and 2020 using the Centers for Disease Control and Prevention (CDC) Wide-ranging Online Data for Epidemiologic Research (CDC WONDER) Multiple Cause of Death (MCOD) database. Secondary objectives were to examine geographic and demographic variations and identify population-level predictors of infection-related mortality.

## Materials and methods

Study design and data source

A retrospective, population-based ecological study was conducted using mortality data from the CDC WONDER Multiple Cause of Death database (CDC WONDER-MCOD) [7]. The MCOD database contains nationwide mortality data collected from U.S. death certificates filed by the National Center for Health Statistics (NCHS) under the National Vital Statistics System (NVSS). Each death certificate contains information about one underlying cause of death, 20 multiple (contributing) causes of death, and the demographics of the deceased individual. The database provides annual death counts, crude mortality rates, age-adjusted mortality rates (AAMRs), and corresponding population estimates for population-based epidemiological analyses.

Study population

Mortality data from January 1999 to December 2020 were obtained from the CDC WONDER MCOD database. Records were identified by selecting deaths with malignant neoplasms (International Classification of Diseases, Tenth Revision (ICD-10) codes C00-C97) as the underlying cause of death. These records were further restricted to those listing septicemia (ICD-10 codes A40-A41) and/or influenza and pneumonia (ICD-10 codes J09-J18) as multiple (contributing) causes of death. Data were extracted directly from the CDC WONDER interface, and only aggregated, de-identified mortality data were analysed. Septicemia and influenza/pneumonia infections were chosen because they are among the most frequent and significant infections associated with morbidity and mortality in patients with malignancies. The final study cohort consisted of 863,441 death records. A total of 11,781,428 cancer-related deaths were excluded because they did not have either of the selected infection causes listed among the multiple causes of death.

Variables

The demographic variables included were age group, sex, and race. The geographic variables comprised the U.S. Census region and the 2013 National Center for Health Statistics Urban-Rural Classification Scheme for Counties from the CDC WONDER database. The mortality variables were annual deaths, crude mortality rate, and AAMR. The variables related to the causes of death were malignant neoplasms (ICD-10: C00-C97) as the underlying cause of death, and septicemia (ICD-10: A40-A41) and influenza and pneumonia (ICD-10: J09-J18) as the multiple (contributing) causes of death.

Outcome measures

The primary outcome measure was infection-related mortality among decedents whose underlying cause of death was a malignant neoplasm. Infection was defined by the diagnosis of septicemia and/or influenza and pneumonia as multiple causes of death. Secondary outcome measures included annual mortality burden, crude and AAMR(s), time trends, regional and urban-rural differences, disparities across demographic groups, and independent determinants of infection-related mortality.

Statistical analysis

Descriptive statistics were employed to describe the number of deaths in each year, crude mortality rates, and AAMR(s), both overall and stratified by demographics and geography. The mortality rates were given per 100,000 inhabitants. The temporal trend of AAMR(s) was assessed using the Joinpoint Regression Program version 6.0.1 (Statistical Methodology and Applications Branch, Surveillance Research Program, National Cancer Institute, Bethesda, MD, USA). The Annual Percent Change (APC) and Average Annual Percent Change (AAPC), together with their 95% confidence intervals (CIs), were calculated. Comparisons between two independent groups were performed using the Mann-Whitney U test, while comparisons among three or more groups were conducted using the Kruskal-Wallis test, as the mortality rate distributions were not normally distributed. The independent variables related to infection-associated mortality rates were examined with multivariable negative binomial regression analysis, with the outcome measure being infection-related mortality and the natural logarithm of the population serving as the offset. Negative binomial regression was selected because mortality count data are typically overdispersed, making it more appropriate than Poisson regression. The natural logarithm of the population was included as an offset to account for differences in population size across strata. The results were presented as incidence rate ratios (IRRs) along with 95% CIs. A p-value < 0.05 was deemed statistically significant. All statistical analyses, except Joinpoint regression, were performed using R statistical software version 4.6.0 (R Foundation for Statistical Computing, Vienna, Austria).

Ethical considerations

The study used publicly available, de-identified mortality data obtained from the CDC WONDER database. The dataset contains no identifiable individual-level information and involved no direct participant contact or intervention. Therefore, institutional review board approval and informed consent were not required because the study analysed publicly available, anonymised data.

## Results

Differences in infection-related mortality were evident in all four U.S. Census regions (Table 1). Mortality from infections was highest in the Southern region (323,185 deaths), followed by the Midwestern (184,845), Western (184,616), and Northern regions (170,795). The highest mean (AAMR) was observed in the Northern and Southern regions (12.0 per 100,000 population), followed by the Western and Midwestern regions (11.8 and 11.3 per 100,000, respectively).

**Table 1:** Regional distribution of infection-related mortality AAMR: age-adjusted mortality rate.

Census Region	Total Deaths	Mean AAMR (per 100,000)
Northeast	170,795	12
Midwest	184,845	11.3
South	323,185	12
West	184,616	11.8

**Figure 1:**
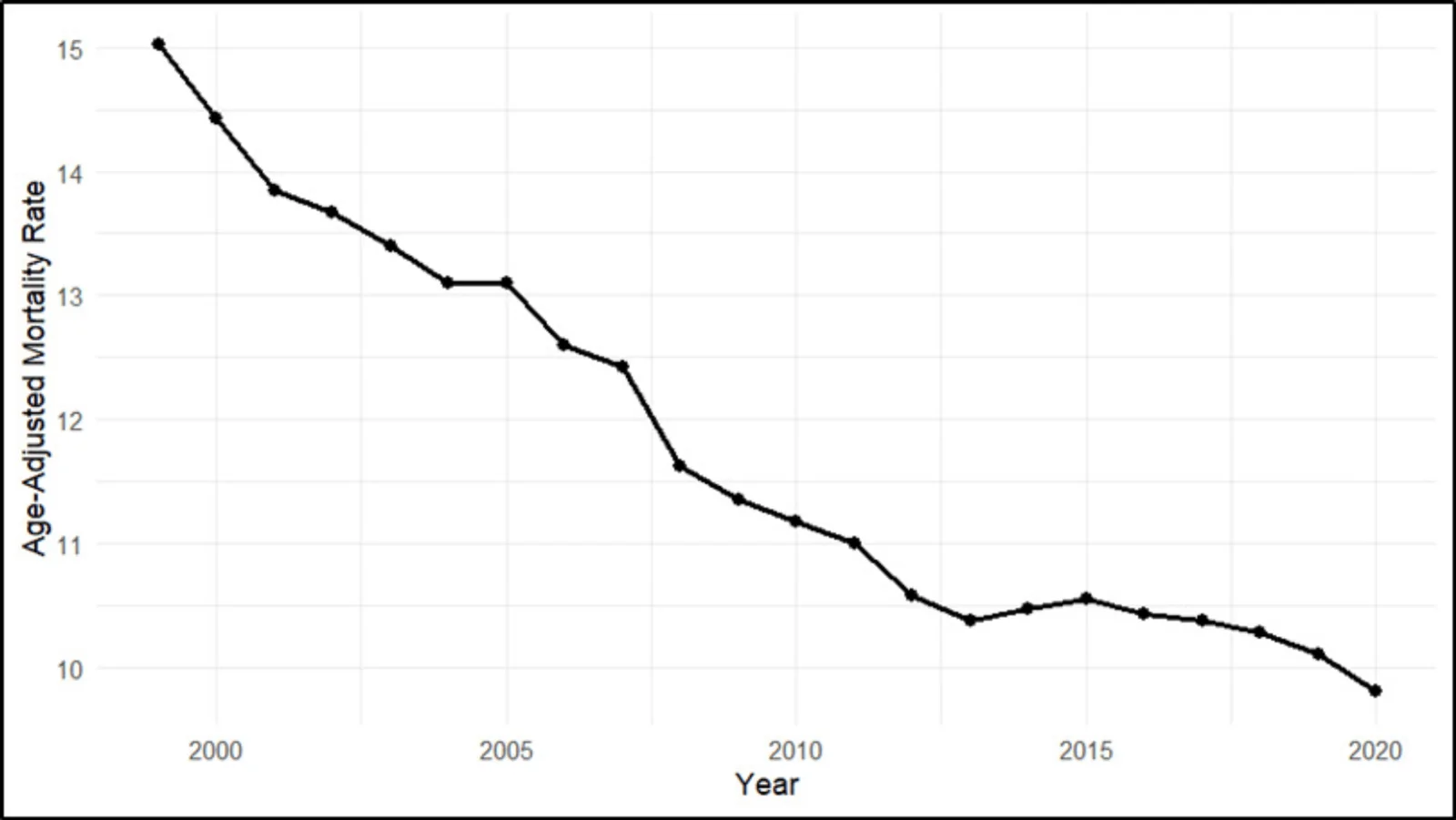
Temporal trends in infection-related mortality Age-adjusted mortality rates are expressed per 100,000 population. The figure illustrates the annual trend in age-adjusted infection-related mortality among patients with underlying malignant neoplasms in the United States from 1999 to 2020.

The temporal pattern of mortality related to infections is given in Table 2 and Figure 1. During 1999-2003, 196,301 infection-related deaths occurred; then 192,935 during 2004-2008; 186,280 during 2009-2013; 202,061 during 2014-2018; and 83,654 during 2019-2020. The highest mean AAMR was observed during 1999-2003 (14.1 per 100,000 population), which gradually declined to 10.0 per 100,000 population during 2019-2020.

**Table 2:** Temporal trends in infection-related age-adjusted mortality AAMR: age-adjusted mortality rate.

Time Period	Total Deaths	Mean AAMR (per 100,000)
1999–2003	196,301	14.1
2004–2008	192,935	12.6
2009–2013	186,280	10.9
2014–2018	202,061	10.4
2019–2020	83,654	10.0

The highest number of infection-related deaths occurred among White individuals (713,804), followed by Black or African American individuals (115,404), Asian or Pacific Islanders (29,353), and American Indian or Alaska Native individuals (4,880). The highest mean AAMR was among Black or African American individuals (16.1 per 100,000 population), followed by White individuals (11.4 per 100,000), Asian or Pacific Islander individuals (10.4 per 100,000), and American Indian or Alaska Native individuals (9.23 per 100,000). The difference in AAMR(s) across racial groups was statistically significant (Kruskal-Wallis χ² = 55.988, df = 3, p < 0.001) (Table 3).

**Table 3:** Comparison of age-adjusted mortality rates across racial groups AAMR: age-adjusted mortality rate.

Race	Total Deaths	Mean AAMR (per 100,000)
American Indian or Alaska Native	4,880	9.23
Asian or Pacific Islander	29,353	10.4
Black or African American	115,404	16.1
White	713,804	11.4

The sex-based differences in infection-related mortality are given in Table 4. More males died from infections (489,410) than females (373,555). In addition, the mean AAMR was higher in males (17.2 per 100,000 population) than in females (9.98 per 100,000 population). The difference in AAMR(s) between the sexes was statistically significant (Mann-Whitney U test, W = 15,172, p < 0.001) (Table 4).

**Table 4:** Sex-based differences in infection-related and age-adjusted mortality rates AAMR: age-adjusted mortality rate.

Sex	Total Deaths	Mean AAMR (per 100,000)
Female	373,555	9.98
Male	489,410	17.2

Urban-rural differences in infection-related mortality are shown in Table 5. The largest number of deaths from infections was recorded in the Large Central Metropolitan area (253,283), followed by the Large Fringe Metropolitan (197,519) and Medium Metropolitan (172,831) areas. The highest AAMR(s), however, were observed in NonCore (Nonmetro) (15.7 per 100,000 population) and Micropolitan (Nonmetro) (15.1 per 100,000 population) areas, while the lowest was in Large Fringe Metropolitan (12.1 per 100,000 population) areas. The differences in AAMR(s) between different categories of urbanisation were statistically significant (Kruskal-Wallis χ² = 53.102, df = 5, p < 0.001).

**Table 5:** Urban–rural disparities in infection-related mortality and age-adjusted mortality rates AAMR: age-adjusted mortality rate; Nonmetro: nonmetropolitan.

2013 Urbanization Category	Total Deaths	Mean AAMR (per 100,000)
Large Central Metro	253,283	13.6
Large Fringe Metro	197,519	12.1
Medium Metro	172,831	12.3
Micropolitan (Nonmetro)	87,996	15.1
NonCore (Nonmetro)	71,503	15.7
Small Metro	79,833	13.3

Infection-associated crude mortality rates were found to differ significantly between the various age groups (Table 6). The number of deaths associated with infections increased from 855 among individuals in the age group 15-20 years to 189,037 among those in the age group 61-69 years. Similarly, the mean crude mortality rates also increased with age, from 0.56 per 100,000 population in the age group 15-20 years to 35.30 per 100,000 population in the age group 61-69 years. The difference in the mortality rates among the different age groups was found to be statistically significant (Kruskal-Wallis χ² = 1047.3, df = 5, p < 0.001).

**Table 6:** Crude infection-related mortality across age groups

Age Group (years)	Total Deaths	Mean Crude Mortality Rate (per 100,000)
15–20	855	0.56
21–30	5,925	0.69
31–40	13,111	1.42
41–50	42,344	4.51
51–60	121,140	14.60
61–69	189,037	35.30

The association between infection-associated mortality rates and infection types is presented in Table 7. During the study period, the total number of deaths related to septicemia (ICD-10: A40-A41) was 500,340, and the number of deaths associated with influenza and pneumonia (ICD-10: J09-J18) was 455,101. The mean AAMR was 7.42 per 100,000 population for septicemia and 6.75 per 100,000 population for influenza and pneumonia.

**Table 7:** Comparison of septicemia and influenza/pneumonia mortality burden AAMR: age-adjusted mortality rate; A40–A41: International Classification of Diseases, Tenth Revision codes for septicemia; J09–J18: International Classification of Diseases, Tenth Revision codes for influenza and pneumonia; n: number of deaths.

Infection Type	Total Deaths (n)	Mean AAMR
Septicemia (A40–A41)	500,340	7.42
Influenza/Pneumonia (J09–J18)	455,101	6.75

The race-wise distribution of deaths from septicemia and influenza/pneumonia is shown in Table 8. White individuals had the highest number of deaths for both infections, with 422,973 deaths due to septicemia and 364,918 deaths due to influenza/pneumonia. On the other hand, Black or African American individuals had the highest mean AAMR in both cases, at 8.63 per 100,000 population for septicemia and 9.12 per 100,000 population for influenza/pneumonia. Asian or Pacific Islander individuals had the lowest mean AAMR for both infections, while American Indian or Alaska Native individuals had intermediate mortality rates.

**Table 8:** Race-wise distribution of septicemia- and influenza/pneumonia-related mortality among patients with underlying malignancies AAMR: age-adjusted mortality rate.

Race	Septicemia Deaths	Septicemia Mean AAMR	Influenza/Pneumonia Deaths	Influenza/Pneumonia Mean AAMR
White	422,973	7.02	364,918	5.69
Black or African American	58,315	8.63	71,088	9.12
Asian or Pacific Islander	16,443	5.89	16,559	5.2
American Indian or Alaska Native	2,609	7.9	2,536	5.78

The distribution of deaths due to septicemia and influenza/pneumonia on the basis of urbanisation categories is shown in Table 9. The Large Central Metropolitan category had the highest number of deaths for both septicemia and influenza/pneumonia, at 139,581 and 142,609, respectively. However, the highest mean AAMR for septicemia was found in NonCore (Nonmetro) (9.17 per 100,000 population) and Micropolitan (Nonmetro) (8.28 per 100,000 population). Similarly, the highest mean AAMR for influenza/pneumonia was found in NonCore (Nonmetro) (7.35 per 100,000 population) and Micropolitan (Nonmetro) (7.24 per 100,000 population) areas.

**Table 9:** Urbanization-wise distribution of septicemia- and influenza/pneumonia-related mortality among patients with underlying malignancies AAMR: age-adjusted mortality rate; Nonmetro: nonmetropolitan.

Urbanization Category	Septicemia Deaths	Septicemia Mean AAMR	Influenza/Pneumonia Deaths	Influenza/Pneumonia Mean AAMR
Large Central Metro	139,581	7.12	142,609	7.12
Large Fringe Metro	111,830	6.24	106,068	6.3
Medium Metro	99,754	6.58	91,734	6.22
Small Metro	48,564	7.16	39,512	6.34
Micropolitan (Nonmetro)	54,958	8.28	42,192	7.24
NonCore (Nonmetro)	45,653	9.17	32,986	7.35

The independent demographic and geographic variables that were significantly associated with infection-related mortality are shown in Table 10. In comparison to American Indian or Alaska Native individuals, higher infection-related mortality rates were observed among White individuals (IRR = 2.41, 95% CI: 2.32-2.50, p < 0.001), Black or African American individuals (IRR = 2.21, 95% CI: 2.13-2.29, p < 0.001), and Asian or Pacific Islander individuals (IRR = 1.39, 95% CI: 1.34-1.45, p < 0.001). Males had higher infection-related mortality than females (IRR = 1.34, 95% CI: 1.31-1.36, p < 0.001). In comparison to Large Central Metropolitan areas, higher infection-related mortality rates were observed in NonCore (Nonmetro) areas (IRR = 1.33, 95% CI: 1.28-1.37, p < 0.001) and Micropolitan (Nonmetro) areas (IRR = 1.15, 95% CI: 1.12-1.19, p < 0.001), while lower infection-related mortality rates were observed in Large Fringe Metropolitan areas (IRR = 0.84, 95% CI: 0.81-0.86, p < 0.001), Medium Metropolitan areas (IRR = 0.94, 95% CI: 0.91-0.96, p < 0.001), and Small Metropolitan areas (IRR = 0.91, 95% CI: 0.84-0.94, p < 0.001).

**Table 10:** Multivariable negative binomial regression analysis of factors associated with infection-related mortality among patients with underlying malignant neoplasms IRR: incidence rate ratio; CI: confidence interval; Nonmetro: nonmetropolitan.

Variable	IRR	95% CI	p-value
Asian/Pacific Islander	1.39	1.34–1.45	< 0.001
Black or African American	2.21	2.13–2.29	< 0.001
White	2.41	2.32–2.50	< 0.001
Male	1.34	1.31–1.36	< 0.001
Large Fringe Metro	0.84	0.81–0.86	< 0.001
Medium Metro	0.94	0.91–0.96	< 0.001
Micropolitan (Nonmetro)	1.15	1.12–1.19	< 0.001
NonCore (Nonmetro)	1.33	1.28–1.37	< 0.001
Small Metro	0.91	0.84–0.94	< 0.001

**Figure 2:**
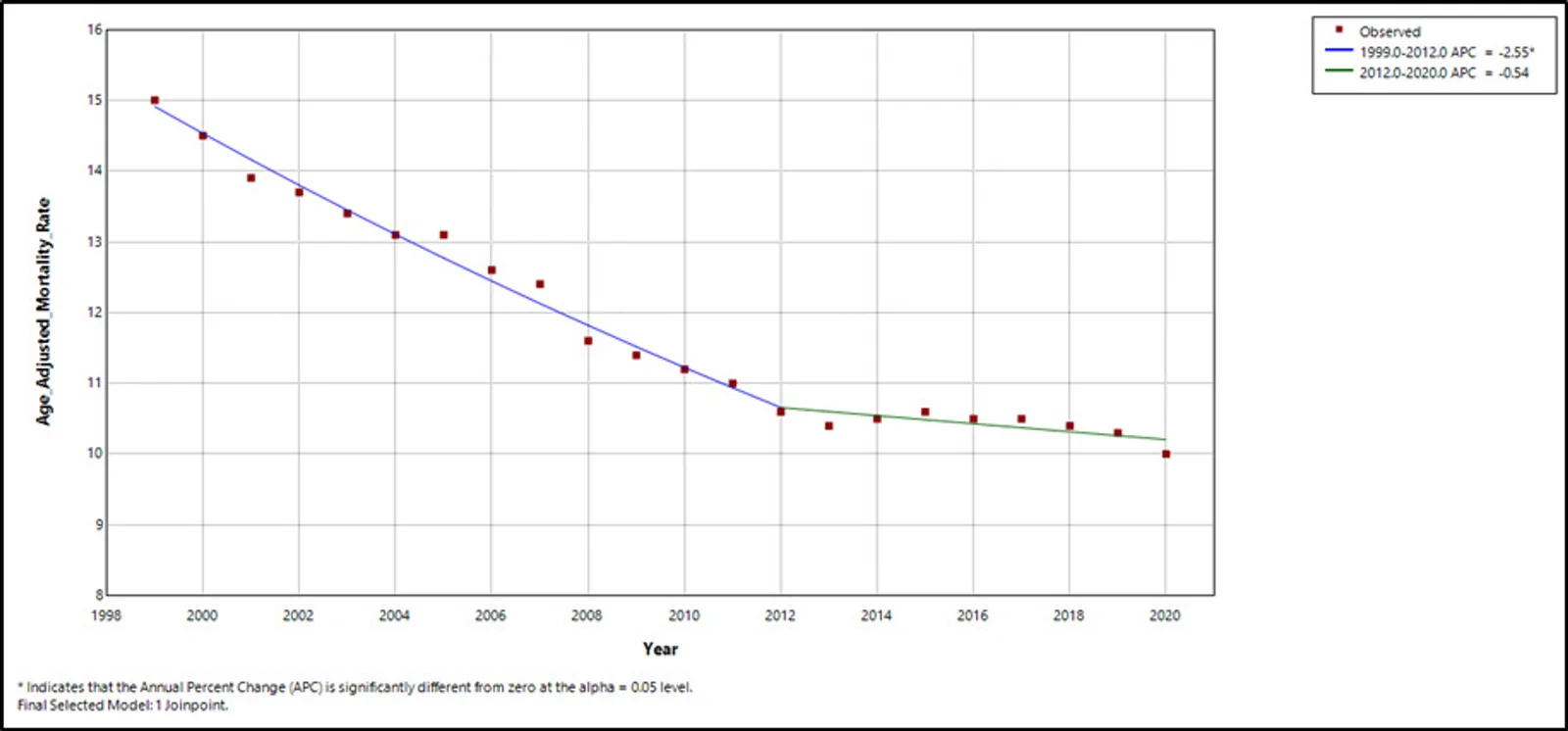
Joinpoint regression of infection-related mortality AAMR: age-adjusted mortality rate; APC: annual percent change. Squares represent the observed annual AAMR(s), while the solid lines represent the fitted Joinpoint regression segments. The asterisk (*) indicates that the APC is significantly different from zero at the α = 0.05 level.

**Figure 3:**
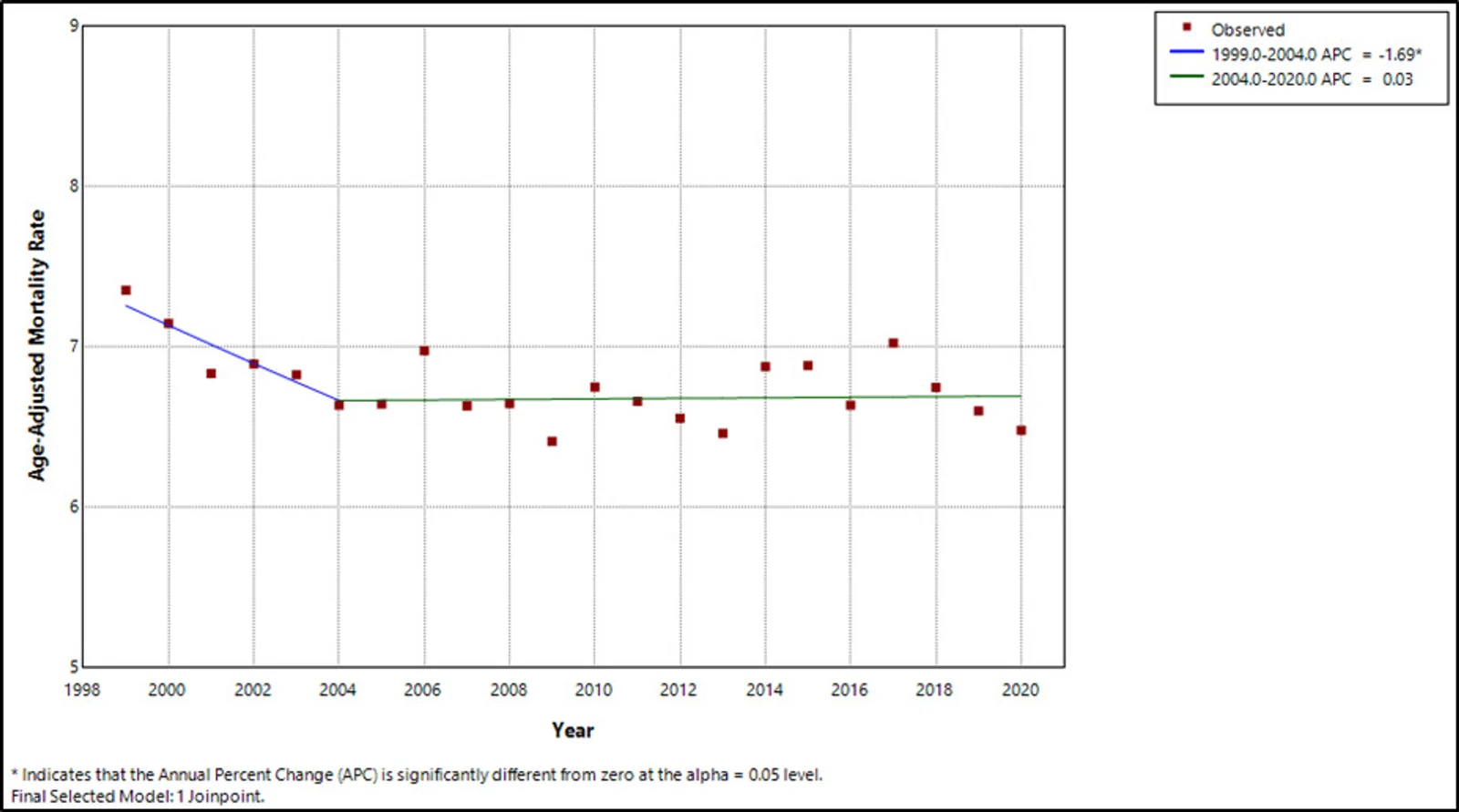
Joinpoint regression analysis of septicemia-related mortality AAMR: age-adjusted mortality rate; APC: annual percent change. Squares represent the observed annual AAMR(s), while the solid lines represent the fitted Joinpoint regression segments. The asterisk (*) indicates that the APC is significantly different from zero at the α = 0.05 level.

**Figure 4:**
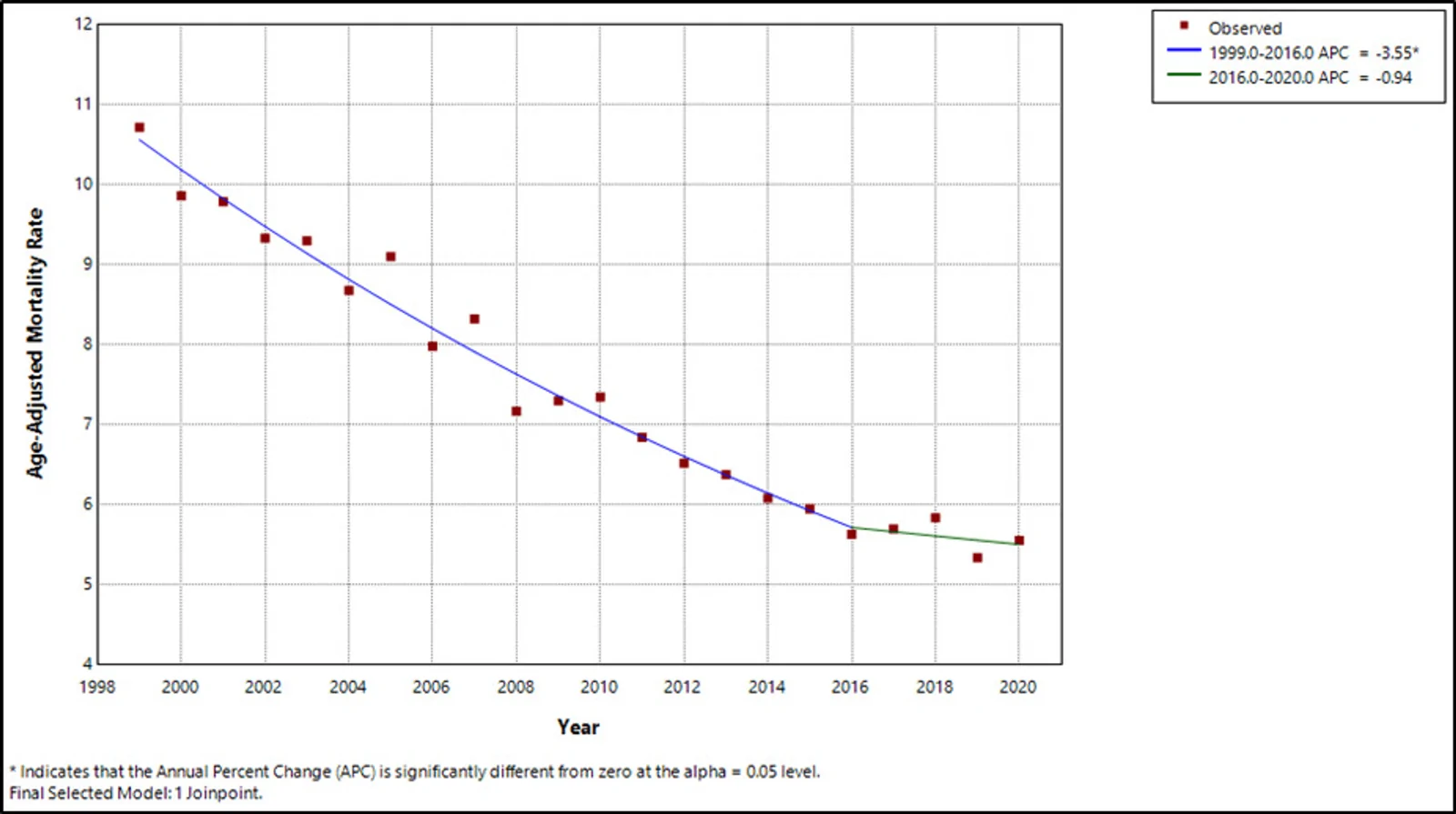
Joinpoint regression analysis of influenza/pneumonia-related mortality AAMR: age-adjusted mortality rate; APC: annual percent change. Squares represent the observed annual AAMR(s), while the solid lines represent the fitted Joinpoint regression segments. The asterisk (*) indicates that the APC is significantly different from zero at the α = 0.05 level.

Infection-related mortality trends have been summarised in Table 11 and Figures 2-4. In general, overall infection-related mortality had a declining trend during the study period, and a joinpoint was recorded in 2012. The annual percent change decreased from -2.53% before 2012 to -0.78% after 2012, with an overall Average Annual Percent Change (AAPC) of -1.87% (95% CI: -2.04 to -1.71; p < 0.001). Septicemia-related mortality had a joinpoint in 2004, with an APC of -1.69% before 2004 and 0.03% after 2004, with an AAPC of -0.38% (95% CI: -0.63 to -0.07; p = 0.018). Influenza/pneumonia-related mortality had a joinpoint in 2016, with an APC of -3.55% before 2016 and −0.94% thereafter, and an AAPC of −3.05% (95% CI: −3.41 to −2.75; p < 0.001).

**Table 11:** Joinpoint regression analysis of temporal trends in infection-related, septicemia-, and influenza/pneumonia-related mortality among patients with underlying malignancies APC: annual percent change; AAPC: average annual percent change; CI: confidence interval; A40–A41: International Classification of Diseases, Tenth Revision codes for septicemia; J09–J18: International Classification of Diseases, Tenth Revision codes for influenza and pneumonia.

Outcome	Joinpoint Year	APC Before Joinpoint (%)	APC After Joinpoint (%)	AAPC (%)	95% CI	p-value
Infection-related mortality	2012	−2.53	−0.78	−1.87	−2.04 to −1.71	< 0.001
Septicemia (A40–A41)	2004	−1.69	0.03	−0.38	−0.63 to −0.07	0.018
Influenza/Pneumonia (J09–J18)	2016	−3.55	−0.94	−3.05	−3.41 to −2.75	< 0.001

## Discussion

This nationwide population-based study of U.S. deaths from 1999 to 2020 associated with infection-related diseases, such as septicemia and influenza/pneumonia, showed a significant impact of both diseases on the U.S. population. The age-adjusted infection-related mortality rate reduced substantially over the period of the study, but the rate has slowed since 2012 and has not changed significantly since 2004 in the case of septicemia-related mortality. There were significant demographic and geographic differences, including the fact that older adults, males, Black or African American individuals, and residents of nonmetropolitan areas were at higher risk for death. Race, male sex, and urbanisation category were also independent predictors of infection-related mortality in a multivariable analysis. Together, these results bring a national perspective on the mortality due to infections in patients with malignancies and offer insights into the population that might benefit from specific and targeted infection prevention and supportive oncology measures.

The severity of the infection-related deaths that were noted is similar to previous population-based data that provide evidence of infectious complications being a major cause of death, other than cancer, in patients with cancer. In a large Surveillance, Epidemiology, and End Results (SEER) linked population-based study, Zheng et al. reported that the most common cancer-specific causes of mortality from infection were septicemia and pneumonia, with the former having over a twofold greater mortality risk compared with the general population [6]. This increased susceptibility to infection is well documented and is due to treatment-induced neutropenia, disruption of mucosal barriers, impaired innate and adaptive immunity, and the use of indwelling vascular devices, which all provide an environment permissive to the invasion of bacteria and respiratory pathogens [3,5]. Nates et al. also noted that, in an immunocompromised cancer population, septic shock has a significantly higher case fatality rate than septic shock in the general critically ill population [4].

The present study showed that infection-related mortality among patients with underlying malignant neoplasms declined significantly during the 22-year study period, but the rate of decline slowed after 2012. This overall decrease in mortality is typical of the progress made in supportive oncology care, such as early detection of severe infection, early empiric antimicrobial therapy, and improved management of neutropenic infection, which have been linked to improved cancer patient outcomes [5]. In addition, standardised sepsis care pathways and performance improvement programmes, as recommended by the Surviving Sepsis Campaign, are associated with increased adherence to evidence-based sepsis management and decreased mortality in hospitalised patients with sepsis [8]. While death rates for infections have continued to decrease since 2012, the less dramatic decrease may be due to persistent problems in preventing and controlling severe infections in a growing population of patients undergoing more complex anticancer therapies and prolonged immunosuppressive treatment [5,9].

One of the most clinically informative findings is the divergent temporal trends of the two infection subtypes. Mortality for influenza/pneumonia decreased at an AAPC of -3.05% (95% CI: -3.41 to -2.75), with a joinpoint in 2016; the mortality rates for septicemia had a slight downward trend after 2004 (APC: 0.03%; AAPC: -0.38%). The better result for respiratory infections is in line with the suggested gradual contribution of the influenza and pneumococcal vaccination campaigns in high-risk groups, as recommended by the Infectious Diseases Society of America (IDSA) clinical practice guidelines on immunocompromised hosts [10]. The mortality from septicemia, on the other hand, was not as low as that from influenza/pneumonia. In contrast to influenza, septicemia is a syndrome of bacterial and fungal pathogens, with no single pathogen being responsible for the syndrome, thus reducing the potential for pathogen-specific prevention. In addition, the high mortality rate for bloodstream infection and septicemia, despite advances in supportive care, has been made more difficult because of the increased prevalence of antimicrobial-resistant organisms among immunocompromised patients with malignancies [11,12].

This age-specific crude mortality trend, increasing with age from 0.56 per 100,000 in the age group 15-20 years to 35.30 per 100,000 in the age group 61-69 years, is similar to the progressive increase seen with ageing for other causes of death, such as immunosenescence, increased burden of cancer, and increased burden of comorbidities [3]. The marked disparity in age-adjusted death rates between males and females (17.2 per 100,000 males compared with 9.98 per 100,000 females, p < 0.001) may be due to well-recognised differences in the distribution of cancer type between sexes and in innate immune responses.

Well-recognised differences in immune function and cancer susceptibility between men and women could account for the higher AAMR(s) in males. The immune system of females is stronger than that of males, with reduced susceptibility to bacterial, viral, fungal, and parasitic infections, while males are more susceptible to infectious diseases with a different immune regulation, as reported by Jaillon et al. [13]. Furthermore, Dorak and Karpuzoglu noted that men are consistently more vulnerable to many cancers, which could be explained by differences in immune surveillance, genetic susceptibility, hormonal effects, and other factors in men compared with women, and which might also account for the increased burden of mortality from infections in male cancer patients [14].

Black/African American people had the highest age-standardised mortality rates for septicemia (8.63 deaths per 100,000 population) and also influenza/pneumonia (9.12 deaths per 100,000 population). This result is consistent with earlier research that has shown that racial differences in cancer outcomes continue to occur in the United States. Similarly, in several different types of cancer, Black patients had significantly less likelihood of receiving guideline-concordant treatment than non-Hispanic White patients, and these differences were associated with greater all-cause and cancer-specific mortality [15]. Likewise, the American Cancer Society (ACS) 2023 report by Islami et al. found that cancer incidence and death rates remain higher among Black people compared with White people, highlighting the continued inequities in access to cancer prevention, early detection, and quality cancer care, which are closely tied to social determinants of health [16].

AAMR(s) are higher in NonCore nonmetropolitan (15.7 per 100,000) and Micropolitan (15.1 per 100,000) areas than in Large Fringe Metropolitan areas (12.1 per 100,000), which follows well-documented rural health disparities. In a national analysis based on the CDC, Henley et al. reported higher cancer mortality in rural areas due to delayed diagnosis, lower specialist density, and lower uptake of cancer prevention services [17]. There are compound disadvantages to patients with cancer in the rural setting in the oncology-infection arena: the lack of access to comprehensive cancer centres, limited access to infectious disease consultation, lower intensive care capacity, and lower early sepsis recognition [17,18]. Regional variation, including the highest total number of deaths in the South (323,185) and the highest mean age-adjusted rates in both the Northeast (12.0 per 100,000) and the South (12.0 per 100,000), is due to both the prevalence of cancer in the region and the persistent differences in healthcare access and insurance coverage [18].

Male sex and rural residence, including NonCore nonmetropolitan (IRR: 1.34, 95% CI: 1.31-1.36) and Micropolitan areas (IRR: 1.15, 95% CI: 1.12-1.19), were independent predictors of elevated infection-related mortality rates after adjustment for the other factors. The longevity of these associations indicates that geography- and sex-based differences are not completely accounted for by age- and race-stratified differences. Rural-urban differences are often exaggerated by crude rates because they oversimplify the complex co-distribution of correlated risk factors, which are more common in rural areas and further exacerbate crude rural-urban differences [18]. The results indicate that both biological and healthcare system factors may play a role in the disparities found.

The results of these findings have significant implications for clinical practice and public health. The continuing decrease in the death rate due to influenza/pneumococcal pneumonia continues to support the ongoing vaccination recommendations for routine influenza and pneumococcal vaccination as an important part of comprehensive cancer care, with a recommended baseline approach of assessing vaccination status upon diagnosis and improving vaccination before or during treatment, when appropriate [19]. In addition, the ASCO guideline highlights vaccination as an important prevention strategy along the cancer care continuum, including survivorship, especially for those receiving immunosuppressive drugs or hematopoietic stem cell transplantation [19]. The steady decline in sepsis-associated mortality since 2004 has also emphasised the critical need for antimicrobial stewardship and early recognition of sepsis, especially in the oncology setting, given the rise in antimicrobial resistance [11,12]. Additional preventable morbidity and mortality could be reduced in high-risk populations through customised survivorship programmes that include infection risk assessment, vaccination, and patient education about the early recognition of serious infection and sepsis [19]. These findings should be interpreted in the light of the study design, as the study was observational and causal inferences should not be made.

Nationally representative population-level data were available and were generated over 22 years using the CDC WONDER Multiple Cause of Death database, which minimised selection bias from individual institutions. Multiple cause of death data included deaths where the underlying cause was septicemia or influenza/pneumonia plus other causes, giving a more comprehensive picture of the infectious burden than underlying cause-only analyses. Statistically significant inflection points were detected, and the annual change in mortality trends was quantified using Joinpoint regression. A multivariable negative binomial regression, which was suitable for the overdispersed count data, allowed for the simultaneous adjustment of the demographic and geographic covariates.

Limitations

Data on death certificate coding may be subject to misclassification, as accurate recording of septicemia or influenza/pneumonia as contributing causes of death depends on the certifying physician's judgement and coding practices, which may have varied across regions and over time. In addition, temporal changes in death certification and coding practices may have influenced the recording of contributing causes of death, thereby affecting comparisons across the study period. The inclusion of mortality data from the final study year (2020) may also have been influenced by the COVID-19 pandemic, which could have affected infection-related mortality patterns and death certification practices. Causal inferences cannot be drawn because of the ecological observational study design. Important clinical variables, including cancer stage, histological subtype, treatment regimen, and immunosuppressive status, were unavailable in the database and therefore could not be adjusted for. Furthermore, all malignant neoplasms were analysed as a single group; consequently, cancer-specific differences in infection susceptibility and mortality could not be evaluated and may have been obscured. The absence of microbiological data limited the mechanistic interpretation of infection-related mortality patterns. Finally, residual confounding from unmeasured patient-level factors, including socioeconomic status, comorbidities, healthcare access, insurance status, cancer treatment, and disease severity, could not be excluded.

## Conclusions

This nationwide population-based study highlights that infection-related mortality remains an important contributor to mortality among patients with malignant neoplasms in the United States. The findings underscore the importance of continued attention to infection prevention in comprehensive cancer care. These findings support continued emphasis on appropriate vaccination, antimicrobial stewardship, and early recognition and management of severe infections, as well as continued efforts to improve access to supportive oncology care for this vulnerable population. Further patient-level studies are warranted to identify effective strategies for reducing the burden of infection-related mortality.
